# Relative Solvent Accessible Surface Area Predicts Protein Conformational Changes upon Binding

**DOI:** 10.1016/j.str.2011.03.010

**Published:** 2011-06-08

**Authors:** Joseph A. Marsh, Sarah A. Teichmann

**Affiliations:** 1 MRC Laboratory of Molecular Biology, Hills Road, Cambridge CB2 0QH, UK

## Abstract

Protein interactions are often accompanied by significant changes in conformation. We have analyzed the relationships between protein structures and the conformational changes they undergo upon binding. Based upon this, we introduce a simple measure, the relative solvent accessible surface area, which can be used to predict the magnitude of binding-induced conformational changes from the structures of either monomeric proteins or bound subunits. Applying this to a large set of protein complexes suggests that large conformational changes upon binding are common. In addition, we observe considerable enrichment of intrinsically disordered sequences in proteins predicted to undergo large conformational changes. Finally, we demonstrate that the relative solvent accessible surface area of monomeric proteins can be used as a simple proxy for protein flexibility. This reveals a powerful connection between the flexibility of unbound proteins and their binding-induced conformational changes, consistent with the conformational selection model of molecular recognition.

## Graphical abstract

**Graphical Abstract:**

**Figure:**
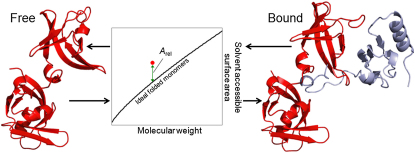

**Highlights:**

► Relative solvent accessible surface area (*A*_rel_) is simple to calculate ► *A*_rel_ predicts conformational changes upon binding from monomers or bound subunits ► Analysis of protein complexes suggests large conformational changes are common ► Intrinsic protein flexibility correlates with conformational changes upon binding

## Introduction

Interactions between polypeptide chains are integral to most biological processes. Our understanding of the molecular mechanisms underlying these interactions has been greatly enhanced by the determination of a large number of three-dimensional structures of proteins in both monomeric and complexed states. This has revealed that protein interactions can be associated with varying amounts of conformational change, from slight shifts in the conformations of side chains to large changes in the relative orientations of domains (Gerstein et al., 1994; Betts and Sternberg, 1999; Janin et al., 2007). Protein interactions can also be accompanied by substantial disorder-to-order transitions in the case of intrinsically disordered proteins (IDPs), which are disordered in isolation but which can often be induced to fold in the presence of binding partners (Wright and Dyson, 2009).

Previously, others have noted a relationship between the conformational changes a protein undergoes upon binding and the interface size of the resulting complex. Janin et al. predicted that complexes with large interfaces would require major structural changes upon binding due to the excessive accessible surface of their isolated subunits (Janin et al., 1988). Later, it was observed that the subunits of protein complexes with large interfaces (>2000 Å^2^) tend to undergo greater conformational changes upon binding than those with smaller interfaces (Lo Conte et al., 1999). Large interfaces relative to protein size have also been noted as a feature of IDPs that fold only upon binding (Gunasekaran et al., 2003, 2004; Levy et al., 2004; Mészáros et al., 2007). However, despite the recognition of this relationship, there have been few analyses of how conformational changes correlate with interface size or other structural features.

The connection between the intrinsic flexibility of unbound proteins and the conformations they adopt upon binding has been the subject of much speculation. In particular, the conformational selection model has gained prominence. This postulates that proteins exist as ensembles of thermally accessible conformations while free in solution, and that binding occurs via transiently formed conformations that resemble the bound state (Tsai et al., 1999). Computational and experimental studies have provided strong evidence that bound-state-like conformations often exist within the free-state ensemble (Boehr et al., 2009; Gsponer et al., 2008; Lange et al., 2008; Marsh et al., 2010; Tobi and Bahar, 2005). While there is some evidence that more flexible proteins tend to undergo larger conformational changes upon binding (Dobbins et al., 2008), little difference was seen between the predicted flexibility of protein binding sites that undergo large conformational changes upon ligand binding and those that do not (Gunasekaran and Nussinov, 2007).

Much work has focused upon trying to predict the structures of protein complexes given the known structures of free subunits, i.e., docking. While current methods have demonstrated a high degree of accuracy for predicting complexes when the subunits maintain conformations close to their free states, docking is much more difficult for proteins that undergo significant conformational changes. Nevertheless, recent approaches incorporating protein flexibility have considerably improved the accuracy of docking (Andrusier et al., 2008; Bonvin, 2006; Wang et al., 2007).

In this study, we start with an approach that may be considered the opposite of docking: given the known structure of a protein complex, we seek to predict the extent of conformational change the constituent subunits undergo upon binding. We show that, while interface size shows some correlation with conformational change, the solvent accessible surface area of a bound subunit relative to the value expected for a monomeric protein of its size is a much better predictor. By applying this method to a large number of protein complexes, we observe that proteins tend to undergo larger conformational changes than would be expected from only considering cases where both monomeric and complex structures are available. In addition, we observe a significant increase in intrinsic disorder in regions predicted to undergo large conformational changes upon binding. Finally, we show that the relative solvent accessible surface area of a bound subunit can be used to predict the likelihood that it is highly flexible or disordered in its monomeric state. Even more interestingly, we find that the relative solvent accessible surface area of monomeric proteins is also useful for predicting their intrinsic flexibility and their expected conformational changes upon binding. This demonstrates a strong relationship between protein flexibility and binding and has immediate practical implications for the modeling of protein complexes.

## Results and Discussion

### Interface Size Shows Limited Correlation with Conformational Changes upon Binding

We searched the Protein Data Bank (PDB) (Berman et al., 2000) for nonredundant pairs of structures where the same or nearly identical polypeptide chains are observed in monomeric and complexed states (see Experimental Procedures). In total, we collected 68 pairs of matching monomer/homomers and 117 pairs of matching monomer/heteromers. Note that since monomeric structures exist, these are transient (as opposed to obligate/permanent complexes, essentially by definition) (Nooren and Thornton, 2003).

**Figure 1:**
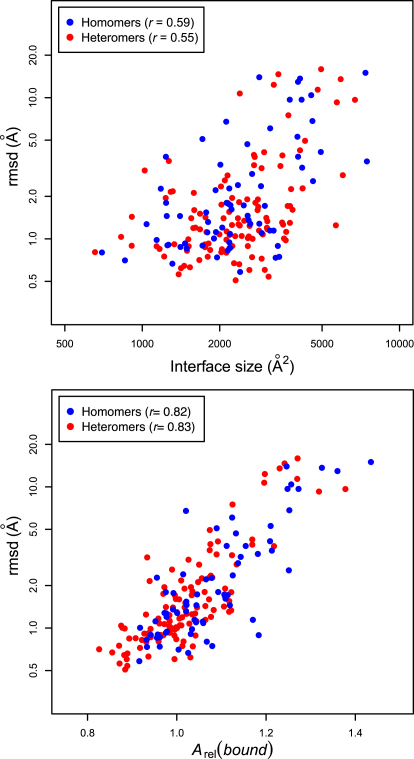
Correlation of Interface Size and Relative Solvent Accessible Surface Area of Bound Subunits (*A*_rel_(*bound*)) with Conformational Changes upon Binding as Given by the rmsd between Free and Bound States Note that rmsd is plotted on a logarithmic axis, consistent with the correlations presented. See also Figures S1 and S2.

In order to assess the relationship between interface size and binding-induced conformational changes, we plotted interface size versus the all-atom root mean square deviation (rmsd) for the full polypeptide chains between bound and unbound conformations for all pairs in the monomer/complex set (Figure 1A). There are only moderate correlations between interface size and log(rmsd) for homomers (*r* = 0.59) and heteromers (*r* = 0.55). While 84% of subunits with rmsd values >2 Å and all subunits with rmsd values >5.1 Å have interfaces >2000 Å^2^ (compared with 67% of all subunits), 62% of subunits with interfaces >2000 Å^2^ have rmsds <2 Å. Thus, it appears that, while a large conformational change is generally associated with the formation of a large interface, a large interface alone is not necessarily a good indicator of conformational change.

One possible reason for the relatively weak correlation between interface size and rmsd is that this analysis only includes the conformational changes of individual subunits, whereas an interface is formed by two or more subunits. For a large interface to form, one can imagine that only a single binding partner would be required to undergo a large conformational change. However, this explanation does not apply to homomers due to their symmetric oligomerization. Since the correlation for homomers is only slightly greater than for heteromers, the contribution of this effect is likely small.

### Relative Solvent Accessible Surface Area of Bound Subunits Is Predictive of Conformational Changes upon Binding

Given the limited correlation between interface size and rmsd, we sought to identify a more useful parameter for predicting conformational changes upon binding. Previous work has shown that the solvent accessible surface area (*A*_s_) of a folded protein can be predicted with high accuracy from its molecular weight using a simple power-law relationship (Miller et al., 1987a, 1987b; Teller, 1976; Janin, 1976). In Figure S1 (available online), we plot molecular weight versus *A*_s_ for 4988 monomeric proteins. The fit to that plot results in Equation 1, where *M* is molecular weight. As expected, there is a strong correspondence between *A*_s_ and *M*, and Equation 1 predicts *A*_s_ with an average absolute deviation of 5.8% for all monomers.

(1) $$A_{s}=4.84M^{0.760}$$

Since Equation 1 is highly predictive of the *A*_s_ of monomeric, folded proteins, we reasoned that the deviation of *A*_s_ from its predicted value could be a useful indicator of to what extent a protein resembles a folded monomer. Thus, we introduce the term relative solvent accessible surface area (*A*_rel_) as the observed *A*_s_ scaled by its predicted value from Equation 1:

(2) $$A_{rel}=\frac{A_{s}^{observed}}{A_{s}^{predicted}}.$$

Importantly, we consider the *A*_rel_ value of each subunit in its bound conformation, but in isolation from the rest of the complex (*A*_rel_(*bound*)). As an isolated subunit with a high *A*_rel_(*bound*) value exposes more surface area than expected for a stable monomeric protein, we predict that significant conformational changes from its monomeric state are likely upon binding. This is similar to the previous observation that the subunits of some protein complexes would expose too much surface if their bound-state conformations were maintained in isolation, implying that intermolecular interactions must be responsible for their stabilization (Miller et al., 1987b).

In Figure 1B, we plot *A*_rel_(*bound*) for each subunit from the monomer/complex data set versus the rmsd between bound and unbound conformations. The agreement is far better than for interface size, with correlations between *A*_rel_(*bound*) and log(rmsd) of 0.82 for homomers and 0.83 for heteromers. If a linear relationship between *A*_rel_(*bound*) and log(rmsd) is assumed, we obtain Equations 3 and 4. These relations allow the rmsds to be predicted with average absolute deviations of 41% for homomers and 34% for heteromers.

(3) $$RMSD_{homomer}=\exp\left(6.14A_{\text{rel}}\left(bound\right)-5.95\right)$$

(4) $$RMSD_{heteromer}=\exp\left(6.35A_{\text{rel}}\left(bound\right)-6.05\right)$$

We can also make more general statements about the relationship between *A*_rel_(*bound*) and rmsd. For example, 79% of subunits with *A*_rel_(*bound*) >1.2 have rmsds >5 Å (compared with 11% of all subunits) and 72% of subunits with *A*_rel_(*bound*) >1.1 have rmsds >2 Å (compared with 30% of all subunits). Conversely, only 16% of subunits with *A*_rel_(*bound*) <1.1 and 6% of subunits with *A*_rel_(*bound*) <1 have rmsds >2 Å. Thus, we suggest that *A*_rel_(*bound*) values >1.2 can be used as strong indicators of large conformational changes upon binding (>5 Å rmsd), while subunits with *A*_rel_(*bound*) values between 1.1 and 1.2 are likely to undergo moderate conformational change (>2 Å rmsd). On the other hand, subunits with *A*_rel_(*bound*) <1.1 and especially <1 are much less likely to undergo significant conformational change.

It could be suggested that the correlation between *A*_rel_(*bound*) and rmsd may arise from the presence of multidomain proteins in the data set. If these proteins have flexible linkers between more globular domains, this may cause these proteins to have larger *A*_rel_(*bound*) values, while changes in the relative orientations of domains may lead to large rmsd values. We have addressed this in Figure S2 by repeating the analysis in Figure 1B using only proteins identified as having a single SCOP domain (Murzin et al., 1995). The correlations in this case are in fact even higher (0.88 for homomers, 0.84 for heteromers), demonstrating that the strong relationship between *A*_rel_(*bound*) and rmsd is not due to flexible interdomain linkers.

### Large Predicted Conformational Changes upon Binding Are Common

**Figure 2:**
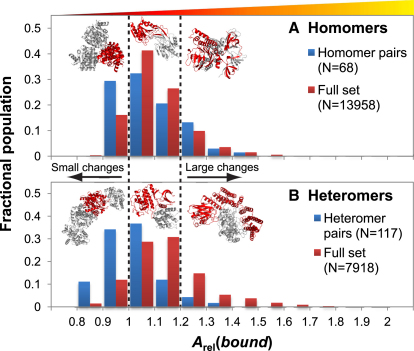
Large Conformational Changes upon Binding Predicted by Large *A*_rel_(*bound*) Values Are Common Distribution of *A*_rel_(*bound*) values from subunits of (A) homomeric and (B) heteromeric complexes from the monomer/complex data set (blue) and from the full set of complexes (red). The complexes above show subunits (highlighted in red) with various *A*_rel_(*bound*) values. In (A), these are 1EKQ (*A*_rel_(*bound*) of 0.96), 1U7Z (1.13) and 3FVQ (1.34). In (B), these are 2CLK (0.94), 1UNL (1.10), and 1DCE (1.32).

Given the apparent utility of *A*_rel_(*bound*) for predicting protein conformational changes upon binding, we investigated the distribution of *A*_rel_(*bound*) values in the full set of protein complexes from the PDB (after filtering, see Experimental Procedures). In Figure 2, we compare the distributions of *A*_rel_(*bound*) values for the full set of complexes to those in the monomer/complex data set. For homomers, there is a slight, albeit not quite significant, increase in the average *A*_rel_(*bound*) value, from 1.08 in the monomer/complex set to 1.10 in the full set of complexes (p = 0.07, Wilcoxon test). For heteromers, however, there is a very large increase in *A*_rel_(*bound*) values, from 1.02 in the monomer/complex set to 1.14 in the full set (p = 2e-23).

The much larger *A*_rel_(*bound*) values in the full set of heteromers compared with the monomer/complex data set suggest that binding-induced conformational changes are greater in general than might be expected from only considering complexes with corresponding monomeric structures available. For example, while only 6% of subunits from the monomer/heteromer set had *A*_rel_(*bound*) values >1.2, more than four times as many (27%) have values that large in the full set of complexes. This suggests that large conformational changes upon binding are common, and that considering only those cases where a monomeric crystal structure is known may lead to bias against large conformational changes in our understanding of molecular recognition mechanisms.

A simple explanation for the increased *A*_rel_(*bound*) values in the full set of protein complexes comes from the hypothesis that the flexibility of unbound proteins correlates with the amount of conformational change they undergo upon binding. Since more flexible proteins are less likely to form diffraction-quality crystals, this would select against large conformational changes in the monomer/complex data set.

### Intrinsically Disordered Proteins in Complex Have Larger A_rel_(bound) Values Than Folded Proteins

Intrinsic protein disorder represents an extreme case of protein flexibility that may partially account for the increased *A*_rel_(*bound*) values in the full set of complexes. Indeed, a tendency for high *A*_s_ values of isolated subunits in their bound conformations was previously noted as a hallmark of IDPs (Gunasekaran et al., 2004), which is very similar to our use of *A*_rel_(*bound*).

**Figure 3:**
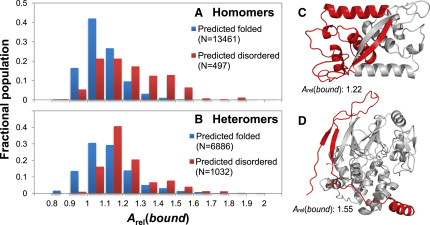
Intrinsically Disordered Proteins in Complex Have Large *A*_rel_(*bound*) Values (A and B) Distribution of *A*_rel_(*bound*) values for (A) homomeric and (B) heteromeric subunits predicted to be intrinsically disordered and predicted to be folded. (C) Homodimeric two-state folder (i.e., unfolded as a monomer) MetJ (A chain, red, B chain gray) (1CMB). (D) PP1-binding domain of spinophilin which has been experimentally characterized as disordered in isolation (red), in complex with PP1 (gray) (3EGG).

To determine to what extent intrinsic disorder contributes to the increased *A*_rel_(*bound*) values observed in the full data set, we used the algorithm FoldIndex (Prilusky et al., 2005) to predict whether subunits were likely to be intrinsically disordered in isolation. FoldIndex provides a single score for an entire polypeptide chain, with values less than zero indicating that a protein is likely to be disordered. In Figures 3A and 3B, we split the subunits into two groups: predicted to be disordered (FoldIndex score <0) and predicted to be folded (FoldIndex score ≥0). According to this classification, 4% of homomeric subunits and 13% of heteromeric subunits are disordered in isolation. Thus, if IDPs tend to have large *A*_rel_(*bound*) values, this can explain the greater increase in *A*_rel_(*bound*) values in the full data set versus the monomer/complex data set for heteromers compared with homomers.

The average *A*_rel_(*bound*) values are significantly increased for both the disordered homomeric (1.24 versus 1.10, p = 3e-72, Wilcoxon test) and heteromeric (1.22 versus 1.13, p = 4e-79) subunits. Notably, there is a much greater fraction of proteins with very large *A*_rel_(*bound*) values in the disordered subunits. We suggest that these high *A*_rel_(*bound*) values correspond to proteins that are completely disordered in their monomeric forms and which adopt highly extended conformations upon binding. Proteins predicted to be disordered but with lower *A*_rel_(*bound*) values may represent limitations of the prediction method and may not actually be intrinsically disordered. Alternatively, some could be IDPs with significant preformed structure, which might thus undergo smaller conformational changes upon binding.

Figures 3C and 3D show examples of proteins predicted to be intrinsically disordered with high *A*_rel_(*bound*) values. In Figure 3C is homodimeric MetJ (PDB ID: 1CMB). Although the concept of intrinsic disorder is often not applied to homomers, many homomeric complexes are known to undergo two-state folding in which the subunits are unfolded in isolation and only fold upon complex formation (Xu et al., 1998). MetJ is both predicted to be disordered (FoldIndex score of −0.04) and experimentally observed to be a two-state folder (Johnson et al., 1992). These observations are consistent with the fairly large *A*_rel_(*bound*) value observed (1.22).

Figure 3D shows protein phosphatase 1 (PP1) in complex with the PP1-binding domain of spinophilin (PDB ID: 3EGG). This region of spinophilin is known to be highly disordered in isolation (Marsh et al., 2010; Ragusa et al., 2010) and is also predicted to be disordered (FoldIndex score of −0.20). It adopts a highly extended conformation in its complexed state with a very high *A*_rel_(*bound*) value of 1.55, demonstrating the potential utility of this parameter for identifying intrinsically disordered regions.

### Differentiating between Structured and Flexible Binding

**Figure 4:**
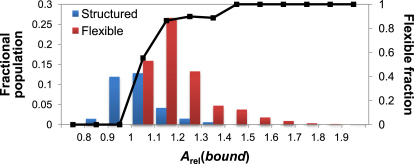
Estimated Distribution Of Heteromeric Subunits from the Full Complex Set Undergoing Structured and Flexible Binding The black line shows the fraction of proteins undergoing flexible binding at different *A*_rel_(*bound*) values and is associated with the right-hand Y axis. See also Figure S3.

While *A*_rel_(*bound*) is useful for predicting the rmsd upon binding for proteins with monomeric crystal structures available, in cases where the unbound state is undergoing large conformational fluctuations (i.e., proteins with high intrinsic flexibility or disorder), its utility is less clear. In other words, the concept of rmsd between two states is very different when one of the states is a dynamic ensemble. Thus, we will make a distinction between “structured” binding, in which the monomeric state adopts a stable, folded structure, and “flexible” binding, in which the monomeric state is intrinsically disordered, unstable or highly flexible. We can make the assumption that the monomer/complex distribution in Figure 2B represents the expected distribution for heteromeric subunits undergoing structured binding since, for these complexes, crystal structures of the monomers are available. The full heteromer data set distribution represents a sum of distributions for structured and flexible binding. If we assume that all of the conformational changes for cases with *A*_rel_(*bound*) <1 are structured, we can split the full data set distribution into structured and flexible components (Figure 4). This allows us to estimate the likelihood that the binding of a subunit with a given *A*_rel_(*bound*) value is either structured or flexible (black line in Figure 4).

By summing the columns in Figure 4, we estimate that 67% of subunits in the full data set undergo flexible binding. We also see that ∼90% of subunits with *A*_rel_(*bound*) >1.2 are predicted to undergo flexible binding. Although considerable assumptions have gone into these estimates, it nevertheless strongly suggests that a large fraction of heteromeric subunits would not be crystallizable in their monomeric state. Therefore, when interpreting *A*_rel_(*bound*) values, one should consider first, the probability that the protein is undergoing structured versus flexible binding, and second, the magnitude of conformational change predicted if the protein is undergoing structured binding.

We note that the 67% of subunits predicted to undergo flexible binding is considerably higher than the 13% predicted to be intrinsically disordered. While this could to some extent reflect limitations in the disorder-prediction method, it is probably primarily due to this group containing many proteins that, while not as flexible as intrinsically disordered proteins, are still too flexible to be crystallized. Thus, this result emphasizes the range of conformational flexibilities proteins can have, rather than only discrete folded and intrinsically disordered states being possible.

### A_rel_ of Free Proteins Correlates with Intrinsic Flexibility and Is Predictive of Conformational Changes upon Binding

*A*_rel_(*bound*) values can only be calculated for complexes with 3D structures available. However, for many complexes, only structures of subunits in their monomeric states currently exist. In this section, we will assess the utility of *A*_rel_ for characterizing the intrinsic flexibility and conformational changes upon binding of monomeric proteins. Just as *A*_rel_(*bound*) values are useful for predicting the conformational changes of bound subunits, we propose that the *A*_rel_ values of free, monomeric proteins (*A*_rel_(*free*)), may be useful for assessing the flexibility of their unbound states. That is, since proteins with higher *A*_rel_(*free*) values expose more surface area and adopt more extended conformations, they are likely to be more flexible.

**Figure 5:**
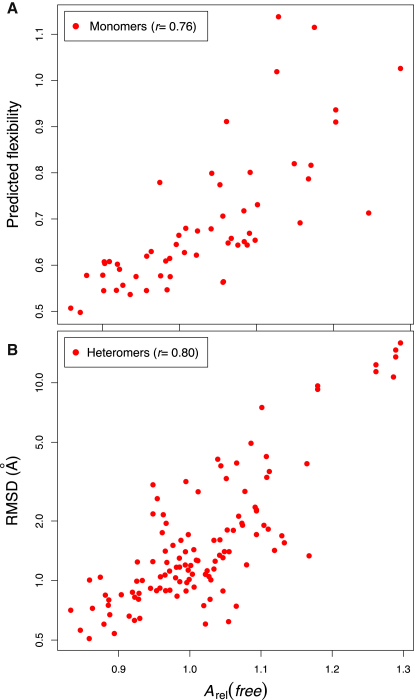
Relative Solvent Accessible Surface Area of Monomeric Proteins (*A*_rel_(*free*)) Correlates with Conformational Changes upon Binding and Intrinsic Flexibility (A) Correlation between *A*_rel_(*free*) and predicted flexibility from normal mode analysis for 60 proteins from Dobbins et al. (2008). Predicted flexibility values are unitless but can be used to compare the relative flexibility of different proteins. (B) Comparison of *A*_rel_(*free*) values for monomeric proteins versus rmsd between unbound and heteromeric conformations. Note that rmsd is plotted on a logarithmic axis, consistent with the correlations presented. See also Figure S4.

In a previous study, Dobbins et al. performed normal mode calculations on a number of unbound proteins and compared them to their conformational changes upon binding (Dobbins et al., 2008). In Figure 5A, we compare the predicted flexibility values from their normal mode calculations against *A*_rel_(*free*) values for 60 monomers in their data set. We observe a significant correlation of 0.76, demonstrating that *A*_rel_(*free*) can indeed be used as a simple means of gauging intrinsic flexibility.

Given this correlation between *A*_rel_(*free*) and flexibility, we can now investigate the relationship between flexibility and binding-induced conformational changes in more detail. In Figure 5B, we have compared *A*_rel_(*free*) versus rmsd for all the monomer/heteromer pairs in our data set and observe a correlation of 0.80, which is only slightly less than was observed for *A*_rel_(*bound*). Thus, the *A*_rel_(*free*) values of unbound subunits appear to have significant utility for predicting conformational changes upon binding. In addition, this provides strong support for the idea that the magnitude of conformational changes upon binding correlates with intrinsic protein flexibility. Assuming a linear relationship between *A*_rel_(*free*) and log(rmsd), we obtain Equation 5, which predicts all rmsds with an average absolute deviation of 37%.

(5) $$RMSD_{heteromer}=\exp\left(6.34A_{\text{rel}}\left(free\right)-6.02\right)$$

As opposed to the strong correlation observed for heteromers, the correlation between *A*_rel_(*free*) and rmsd for homomers is poor (*r* = 0.18, Figure S3). An explanation for this comes from the fact that most of the high *A*_rel_(*bound*) subunits from the homomer data set are domain-swapped dimers (Liu and Eisenberg, 2002). In their domain-swapped conformations, these proteins extend large segments and tend to have high *A*_rel_(*bound*) values, while in their monomeric forms, they resemble normally folded proteins with low *A*_rel_(*free*) values. Thus, the *A*_rel_(*free*) values of monomeric proteins are not useful for predicting the conformational changes they undergo upon self-association.

In Figure S4, we have compared both predicted flexibility values from normal mode calculations and *A*_rel_(*free*) values to rmsd values for the 60 monomer/complex pairs from the study by Dobbins et al. (all complexes from this study were heteromers) (Dobbins et al., 2008). Predicted flexibility values have a correlation of 0.43 with rmsd, confirming the association between flexibility and conformational change previously observed (Dobbins et al., 2008). Notably, the correlation for *A*_rel_(*free*) is even higher (*r* = 0.55). These results strongly support the use of *A*_rel_(*free*) as a simple proxy for the flexibility of free proteins and for predicting the magnitude of binding-induced conformational changes, with *A*_rel_(*free*) being very simple to compute and providing information complementary to normal mode analysis.

One possible way of viewing *A*_rel_ may be that it is, to a certain extent, acting as a measure of protein globularity. Thus, a simple way of interpreting our results is that more globular proteins tend to have less solvent accessible surface area, are less flexible while free in solution, and undergo smaller conformational changes upon binding. Note that this is independent of whether the protein is single or multidomain.

### Examples of Proteins Undergoing Conformational Change

**Figure 6:**
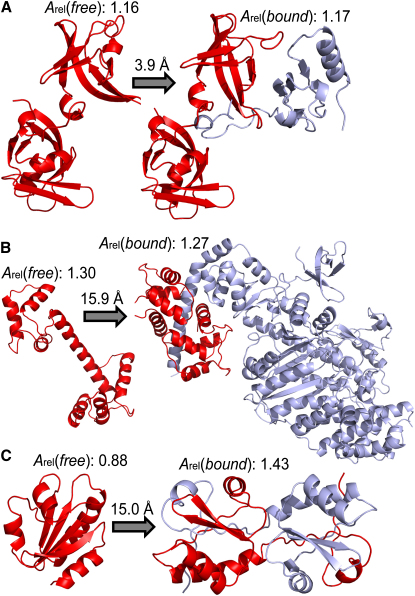
Conformational Changes between Monomeric and Complexed States (A) RimM in its free state (2DYI) and in complex with the ribosomal protein S19 (3A1P). (B) Calmodulin, free (4CLN) and in complex with myosin VI (2VB6). (C) Thioredoxin, monomeric (2O7K) and as a domain-swapped dimer (3DIE).

In Figure 6, we show three examples of proteins in their bound and unbound states in order to illustrate the relationships between *A*_rel_(*bound*), *A*_rel_(*free*) and conformational changes upon binding. Figure 6A shows the 16S ribosomal RNA processing protein RimM in its free state (PDB ID: 2DYI) and in complex with the ribosomal protein S19 (PDB ID: 3A1P). Bound RimM has an *A*_rel_(*bound*) value of 1.17, consistent with the conformational change observed upon binding (rmsd of 3.9 Å), which primarily involves rearrangement of loops and β strands in the one domain that directly interacts with S19. The *A*_rel_(*free*) value of 1.16 indicates significant free-state flexibility that is likely important for facilitating binding.

Figure 6B shows calmodulin (PDB ID: 4CLN) and its complex with myosin VI (PDB ID: 2VB6). Here, we see a large *A*_rel_(*bound*) value (1.27), consistent with the major conformational change upon binding (rmsd of 15.0 Å). Furthermore, calmodulin also has a very large *A*_rel_(*free*) value (1.30), due to its highly extended monomeric structure, implying considerable intrinsic flexibility. Indeed, this is supported by the large number of structures of varying conformations available for both free and bound states of calmodulin, which is likely due to this high flexibility (Chou et al., 2001; Gsponer et al., 2008).

Figure 6C shows thioredoxin in both monomeric (PDB ID: 2O7K) and domain-swapped dimeric states (PDB ID: 3DIE). The *A*_rel_(*bound*) of bound thioredoxin is very large (1.43) and so is the conformational change between monomeric and dimeric states (rmsd = 15.0 Å). However, *A*_rel_(*free*) is quite low (0.88), implying a highly stable monomeric state. This is consistent with the poor correlation between *A*_rel_(*free*) and rmsd mentioned earlier for homomers. In this case, the domain-swapped dimer of thioredoxin only forms upon mutation. Thus, as is probably true for most proteins in the monomer/homomer set, sequence differences and/or differences in sample conditions, and not intrinsic flexibility, account for the large conformational differences between free and bound states.

As an interesting note, during the course of our study, we encountered a pair of structures (monomeric 1CMW and heterodimeric 1BGX) where an *A*_rel_(*bound*) of 1.38 and an *A*_rel_(*free*) of 1.29 predict a very large conformational change upon binding. However, the free and bound states adopted very similar conformations (rmsd of only 1.4 Å) and examination of Figures 1B and 3A shows us that there are no other proteins near these regions of the plots. Further investigation revealed that the monomeric structure 1CMW had been recently retracted from the PDB and is believed to have been fabricated (presumably using the complex structure as a template). Thus, in this case, *A*_rel_ clearly proved its usefulness by identifying an implausible structure.

### Conclusion

In this study, we have presented simple methods for predicting the binding-induced conformational changes of proteins using the relative solvent accessible surface area of either complexed or monomeric structures. Although *A*_rel_(*bound*) and *A*_rel_(*free*) provide no information on the nature of the conformational change, only a predicted magnitude, this will allow expanded data sets and facilitate large-scale analyses of the relationships between protein sequences, structures, flexibility and conformational changes upon binding.

The use of *A*_rel_(*bound*) to predict conformational changes upon binding will be particularly useful for the large number of protein complexes for which no structures of the free subunits have been determined. Thus, one can quickly and easily assess whether an unbound subunit is likely to resemble its bound conformation or whether it would require significant conformational changes. Furthermore, one can consider the likelihood that a bound subunit is highly flexible in its monomeric state: an *A*_rel_(*bound*) value >1.2 indicates a high chance of significant flexibility, while *A*_rel_(*bound*) >1.4 suggests a protein is very likely to be intrinsically disordered. *A*_rel_(*bound*) could also have considerable utility as a structure evaluation tool. For example, in a docking calculation, the predicted complex structure could be compared with the unbound structure to assess whether the amount of conformational change is reasonable.

*A*_rel_(*free*) can be used to predict both the flexibility of free proteins and their conformational changes upon binding. This could also be very useful for the docking of protein complexes: since complexes that require large conformational changes from the monomeric state are much less likely to be successfully modeled, this could allow the probability of a successful docking calculation to be predicted beforehand. In addition, knowledge of the expected conformational change from *A*_rel_(*free*) could also potentially be useful for guiding docking calculations.

There is growing evidence that many proteins can undergo motions over a wide range of length and timescales and that these motions are often important for biological function. Similarly, along with the large body of recent research into the importance of protein disorder has come the awareness of the continuum of protein flexibility and disorder, with some disordered states possessing large amounts of nonrandom secondary and tertiary structure and some folded proteins being highly flexible (Boehr et al., 2009; Gsponer et al., 2008; Jensen et al., 2009; Marsh et al., 2010). Our results support this paradigm by showing that proteins undergo widely varying amounts of conformational change upon binding, and by suggesting that the magnitude of this conformational change tends to correlate with flexibility and intrinsic disorder in the unbound state. Furthermore, a significant correlation between protein flexibility and conformational changes upon binding supports the importance of conformational selection in molecular recognition. The observation that flexible proteins tend to undergo greater conformational changes upon binding suggests that this intrinsic flexibility is important for sampling those conformations resembling the bound state.

Some complexes that lack corresponding monomeric structures are probably obligate (Nooren and Thornton, 2003), i.e., unstable on their own, and thus rarely or never exist in a monomeric state within the cell. Indeed, this could be true for a considerable fraction of the subunits we predicted to undergo flexible binding. For these cases, it might be argued that the ability to predict the conformational changes required of a folded monomeric state would be of limited utility. However, even if a complex is completely obligatory, at some point after polypeptide synthesis the complex must initially assemble and these conformational changes must be important for the assembly process. This does raise the question, however, of how to interpret *A*_rel_(*bound*) values for proteins which undergo significant changes in flexibility upon binding. Can the predicted rmsd values be compared with proteins that undergo structured binding? To what extent do *A*_rel_(*bound*) values correlate with changes in structure and dynamics for flexible proteins?

Since our predictive method is based upon only complexes which have paired monomer crystal structures, it is difficult to assess these questions at this point. Moreover, while the *A*_rel_ method is currently only applicable to crystallizable protein complexes, recent work has demonstrated that some proteins can remain highly flexible or disordered in their complexed states (Fong et al., 2009; Mittag et al., 2010a; Tompa and Fuxreiter, 2008). Such dynamic complexes are largely inaccessible to X-ray crystallography, although they are now in some cases able to be characterized in significant detail by small-angle X-ray scattering and NMR (Marsh et al., 2010; Mittag et al., 2010b; Wells et al., 2008). Future advances in computational and experimental modeling of highly flexible proteins, and especially the development of methods that can be applied on a large scale, will be crucial for fully understanding the range of protein interactions and conformational dynamics utilized within the cell.

## Experimental Procedures

We selected all structures from the full set of protein X-ray crystal structures in the PDB with PISA (Krissinel and Henrick, 2007) quaternary structure assignments determined to a resolution ≤2.5 Å (2010-11-14). Only monomers and subunits longer than 40 residues were considered. Monomers with more than five disordered residues (defined as nonterminal residues not observed in the crystal structure) were ignored. In addition, we filtered out all monomers with ≥25 nonwater HETATM lines in order to remove most monomers with ligands or significant chemical modifications. To ensure that our data set contained only high-confidence monomers, we only considered monomers identified by PISA where both the asymmetric unit and the PDB biological unit contained single chains, and which were not identified as having more than one subunit in the PiQSi database of manually curated quaternary structure (Levy, 2007). We also filtered out protein complexes that had only a single chain in either their asymmetric unit or PDB biological unit, or that were identified as monomers in PiQSi. Complexes containing only a single polypeptide chain longer than 20 residues were removed in order to filter out single protein chains bound to small peptides or nucleic acids. Finally, we removed complexes that were formed by polypeptide cleavage. These were identified as complexes containing unique chains that shared the same UniProt identifiers but which had <90% sequence identity to each other. Together, this filtered set of protein complexes comprised the “full set” referred to in the text.

To generate the monomer/complex pairs data set, a protein BLAST (Altschul et al., 1997) search was performed for all monomers against all unique subunits in multimeric complexes. Only pairs with >98% sequence identity and which differed in molecular weight by less than 1 kDa were retained. Finally, the pairs were filtered so that redundant pairs with >98% sequence identity and similar rmsd values (<1 Å difference) were dropped. The solvent accessible surface area of each structure and isolated subunit was calculated with AREAIMOL (Collaborative Computational Project, 1994). The interface size of each subunit was calculated as the amount of surface area buried between the subunit and the rest of the complex. Complexes were identified as homomeric if all of the protein subunits had ≥90% sequence identify to each other; otherwise they were heteromeric. All pairs from the monomer/complex data set along with their *A*_rel_(*free*), *A*_rel_(*bound*), interface size and rmsd values are provided in Tables S1 and S2.

## Supplemental Information

Document S1. Four Figures

Table S1 contains the homomeric proteins used in our monomer/complex dataset, as well as their associated structural properties relevant to this study

Table S2 is the same as Table S1, but for heteromeric proteins
