# Supplementary material for: Relative Solvent Accessible Surface Area Predicts Protein Conformational Changes upon Binding
Source: Structure. 2011 Jun 8;19(6):859–67. doi: 10.1016/j.str.2011.03.010 (PMC3145976; doi:10.1016/j.str.2011.03.010)
Supplement: Document S1. Four Figures [file mmc1.pdf]

Structure *19*

Supplemental Information

Relative Solvent Accessible Surface Area Predicts Protein Conformational Changes upon Binding

Joseph A. Marsh and Sarah A. Teichmann

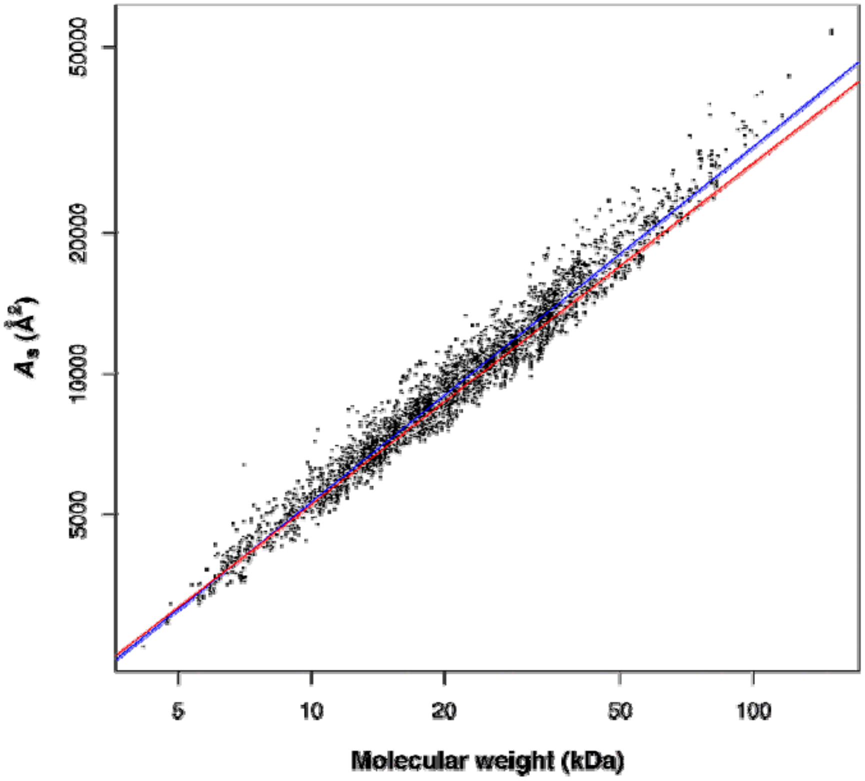

Figure S1, related to Figure 1. Relationship between protein molecular weight and solvent accessible surface area (A_s) for 4988 monomeric proteins. The blue line represents the fit in Equation 1 from this study, while the red line shows the fit derived in Miller et al. (1987a). These fits are very similar at low molecular weights but diverge above ~20 kDa due to the higher molecular weight proteins included in our study.

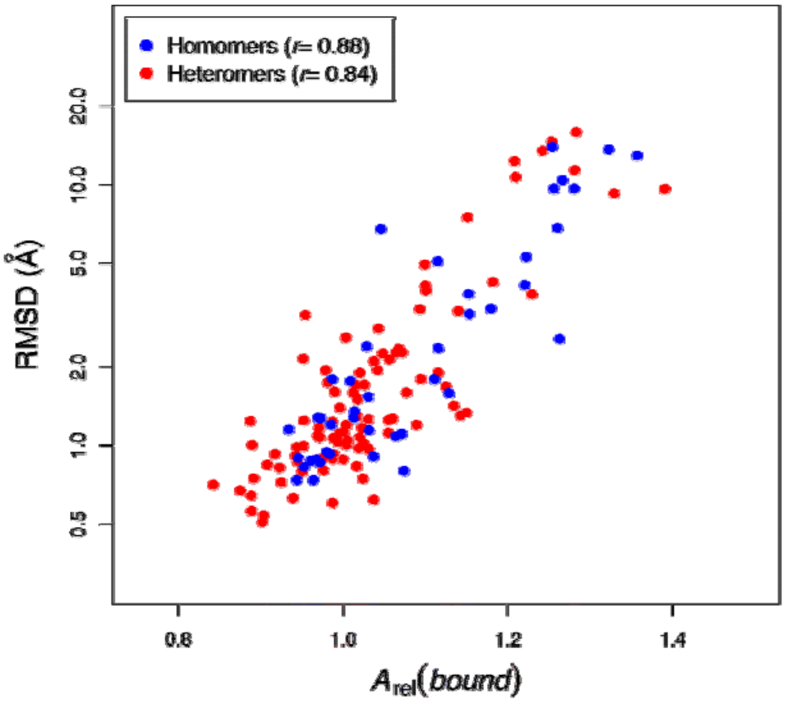

Figure S2, related to Figure 1. Correlation of A_{rel}(*bound*) with RMSD for single-domain proteins as identified in SCOP 1.75 (Murzin et al., 1995).

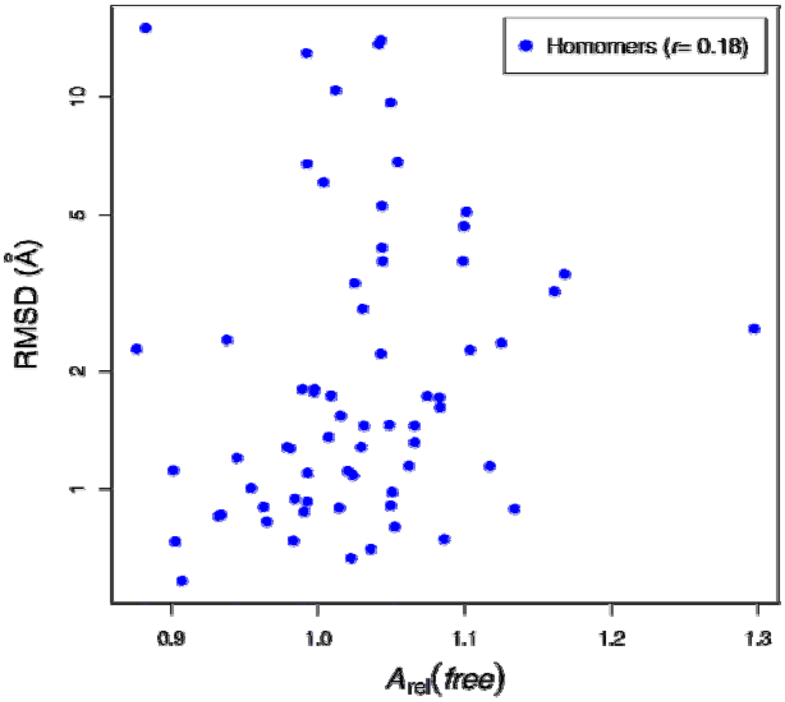

Figure S3, related to Figure 4. Comparison of A_{rel}(*free*) values for monomeric proteins vs. RMSD between unbound and homomeric conformations.

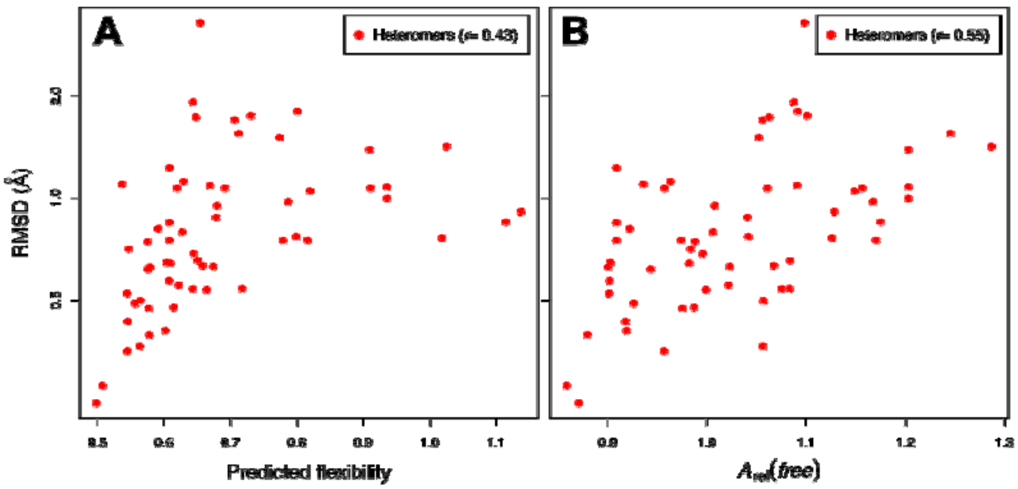

Figure S4, related to Figure 5. *(A)* Correlation between predicted flexibility from normal model analysis and RMSD for 60 monomeric proteins from Dobbins *et al*. *(B)* Correlation between A_{rel}(*free*) and RMSD for the same set of proteins.
